# Giant Choledocholithiasis With Choledochal Cyst: A Report of a Rare Case

**DOI:** 10.7759/cureus.64306

**Published:** 2024-07-11

**Authors:** Vinay V, Nayana S Kumar, Ozair Khan, SK Azharuddin

**Affiliations:** 1 Gastrointestinal Surgery, All India Institute of Medical Sciences, Rishikesh, Rishikesh, IND; 2 General Surgery, All India Institute of Medical Sciences, Rishikesh, Rishikesh, IND; 3 Surgical Gastroenterology, All India Institute of Medical Sciences, Rishikesh, Rishikesh, IND

**Keywords:** mrcp imaging, choledochal cysts, giant choledocholithiasis, cbd stone, gastrosurgery

## Abstract

A giant common bile duct (CBD) calculus is a rare occurrence, and the presence of a giant calculus within a choledochal cyst (CDC) is even more unusual. In this case report, we detail an instance of a giant CBD calculus measuring 7 cm x 3 cm found within a CDC, accompanied by multiple tiny calculi. Magnetic resonance cholangiopancreatography (MRCP) revealed the dilation of the bi-lobar intrahepatic biliary radical (IHBR) and the CBD. A large T2 hypointense and T1 hyperintense calculus occupied the dilated CBD and common hepatic duct (CHD), extending into the left hepatic duct (LHD) and right hepatic duct (RHD). There was a possibility of type 1c CDC with cystolithiasis, hepatolithiasis, and cholelithiasis. The patient underwent open cholecystectomy with choledochotomy, stone retrieval, excision of the CDC, and Roux-en-Y hepaticojejunostomy.

## Introduction

Gallstone disease stands out as one of the most prevalent conditions requiring surgical intervention. Approximately 35% of individuals with gallstones will develop symptoms necessitating cholecystectomy [1]. Stones causing obstruction of the common bile duct (CBD) can originate in the CBD itself, the gallbladder, or the extrahepatic or intrahepatic ducts [2]. Despite advancements, endoscopic retrograde cholangiopancreatography (ERCP) remains the acknowledged "gold standard" for diagnosing pancreatic and biliary ductal pathology [3]. Cystolithiasis and cholecystolithiasis are the prevailing conditions, affecting 70% of adults with choledochal cysts (CDCs). A bile duct calculus is categorized as "large" if it exceeds 1.5 cm and as "giant" when it reaches or exceeds 5 cm in size [2,4-6].

## Case presentation

A 35-year-old female presented to All India Institute of Medical Sciences, Rishikesh surgical gastroenterology OPD with complaints of colicky right upper quadrant pain and discomfort for the past one year, on and off. At presentation, she was not icteric having stable vitals. On clinical examination, the patient had tenderness in the right hypochondrium. Routine blood investigations of hemoglobin, total leucocyte count, and liver function test (LFT) showed total serum bilirubin of 0.64 mg/dL, aspartate transaminase (AST) 18 U/L, and alanine transaminase (ALT) 17 U/L. The renal function test, serum electrolyte, serum glucose, and urine analysis were all normal (Table 1).

**Table 1:** Lab investigation of the patient PT/INR: prothrombin time/international normalized ratio; ALT: alanine transaminase; AST: aspartate transaminase; ALP: alkaline phosphatase; GGT: gamma-glutamyl transferase

Date	At admission	At discharge	Lab reference values
Hemoglobin	10.7	9.2	11.0-13.0 gm%
Total leucocyte count	12.78	12.2	4000-11000/cc
Platelets	122	569	150,000-450,000/cc
PT/INR	12.1/0.97		
Bilirubin (total/direct)	0.80/0.26	0.44/0.17	0.3-1.2 mg/dl
ALT	37	48	0.1-0.3 mg/dl
AST	53	23	0-35 IU
ALP	446	671	30-120 IU
GGT	283	286	0-38 IU
Total protein	8.3	-	6.6-8.3
Albumin	4.2	2.2	3.5-5.2
Globulin	4.1	-	1.5-4.0
Urea/creatinine	20/0.59	31/0.48	17-43/0.55-1.02
Sodium/potassium	137/3.7	131/4.6	135-145/3.5-5.5
anti-HIV/anti-HbsAg/anti-HCV	Non-reactive		

**Figure 1:**
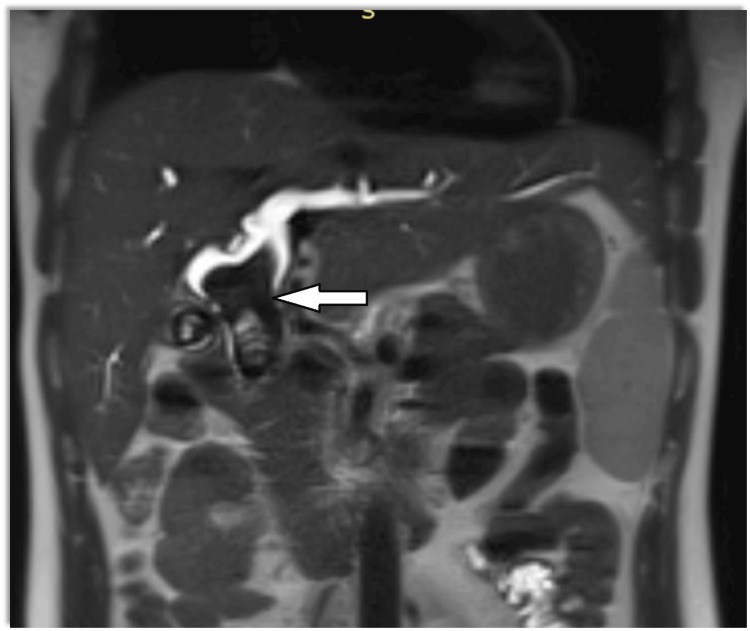
T2 coronal image shows bi-lobar central and peripheral IHBRD and dilated CBD with distal smooth tapering of CBD White solid arrow shows the filling defect. IHBRD: intrahepatic biliary tract dilatation; CBD: common bile duct

**Figure 2:**
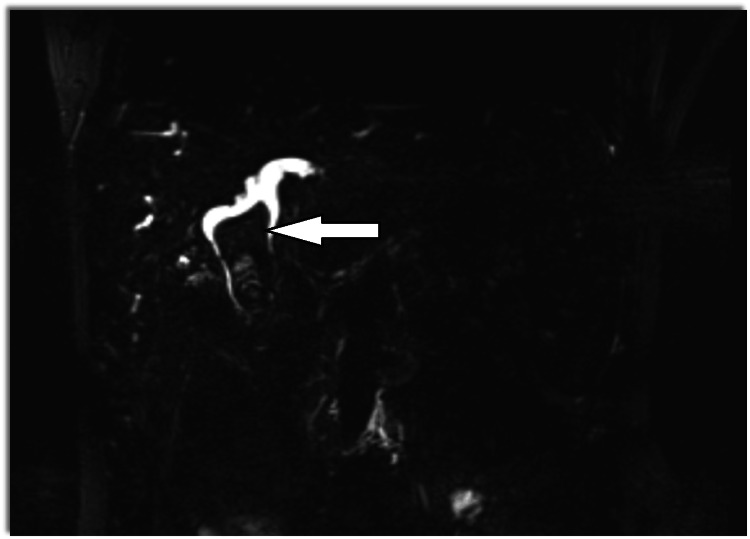
T2 coronal image showing dilated CBD with IHBRD with T1 hypodense CBD stone Solid white arrow showing filling defect in CBD. IHBRD: intrahepatic biliary tract dilatation; CBD: common bile duct

**Figure 3:**
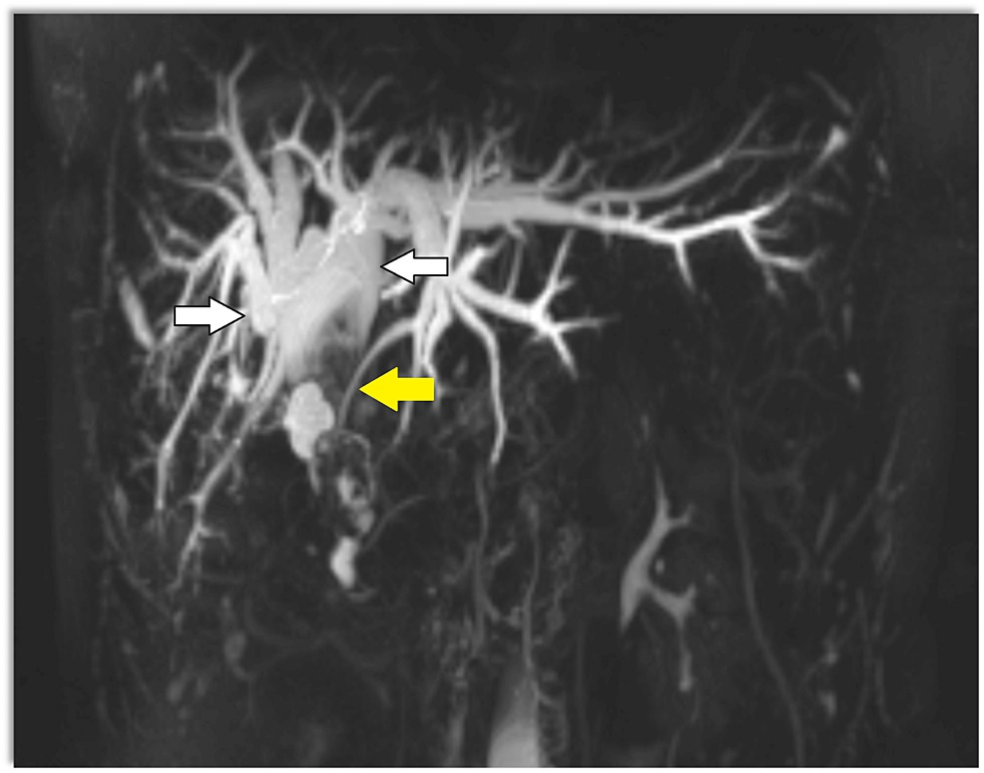
MRCP T2 reconstruction image showing bi-lobar central and peripheral IHBRD and dilated CBD (20 mm) with saccular dilatation, a type 1c choledochal cyst, large T2 hypodense calculus size 6 cm x 2.6 cm is seen White solid arrows show bi-lobar central IHBRD and a yellow solid arrow shows a filling defect. IHBRD: intrahepatic biliary tract dilatation; CBD: common bile duct; MRCP: magnetic resonance cholangiopancreatography

Magnetic resonance cholangiopancreatography (MRCP) showed dilatation of bi-lobar intrahepatic biliary radical (IHBR) with dilatated CBD large T2 hypointense and T1 (Figure 1), hyperintense calculus is seen, occupying dilated CBD and common hepatic duct (CHD). It extends into the left hepatic duct (LHD) and right hepatic duct (RHD), with the possibility of type 1c choledocholithiasis with cystolithiasis, and hepatolithiasis with cholelithiasis (Figures 2, 3).

The patient was planned for elective surgery, intraoperatively gall bladder was distended with calculi and had a thickened and fibrotic wall with Frozen Calot’s triangle. Fusiform dilatation of CBD with distal tapering in intra pancreatic portion (Todani classification, type 1c) filled with giant calculi was seen. There was dense adhesion between the CDC and the portal vein. Stone clearance and intraoperative Cholangioscopy were done. The liver had a micronodular surface with pre-cirrhotic changes. We proceeded to separate the cyst from the proper hepatic artery, right hepatic artery (anteromedially), and portal vein (posteriorly). The gall bladder was dissected out from the gall bladder fossa using electrocautery via the Fundus first approach. The cystic duct was ligated and transected and the gall bladder specimen was taken out. Due to dense adhesions present between the CDC and the portal vein, sub-serosal dissection of the CDC was done to prevent injury to the portal vein posteriorly.

**Figure 4:**
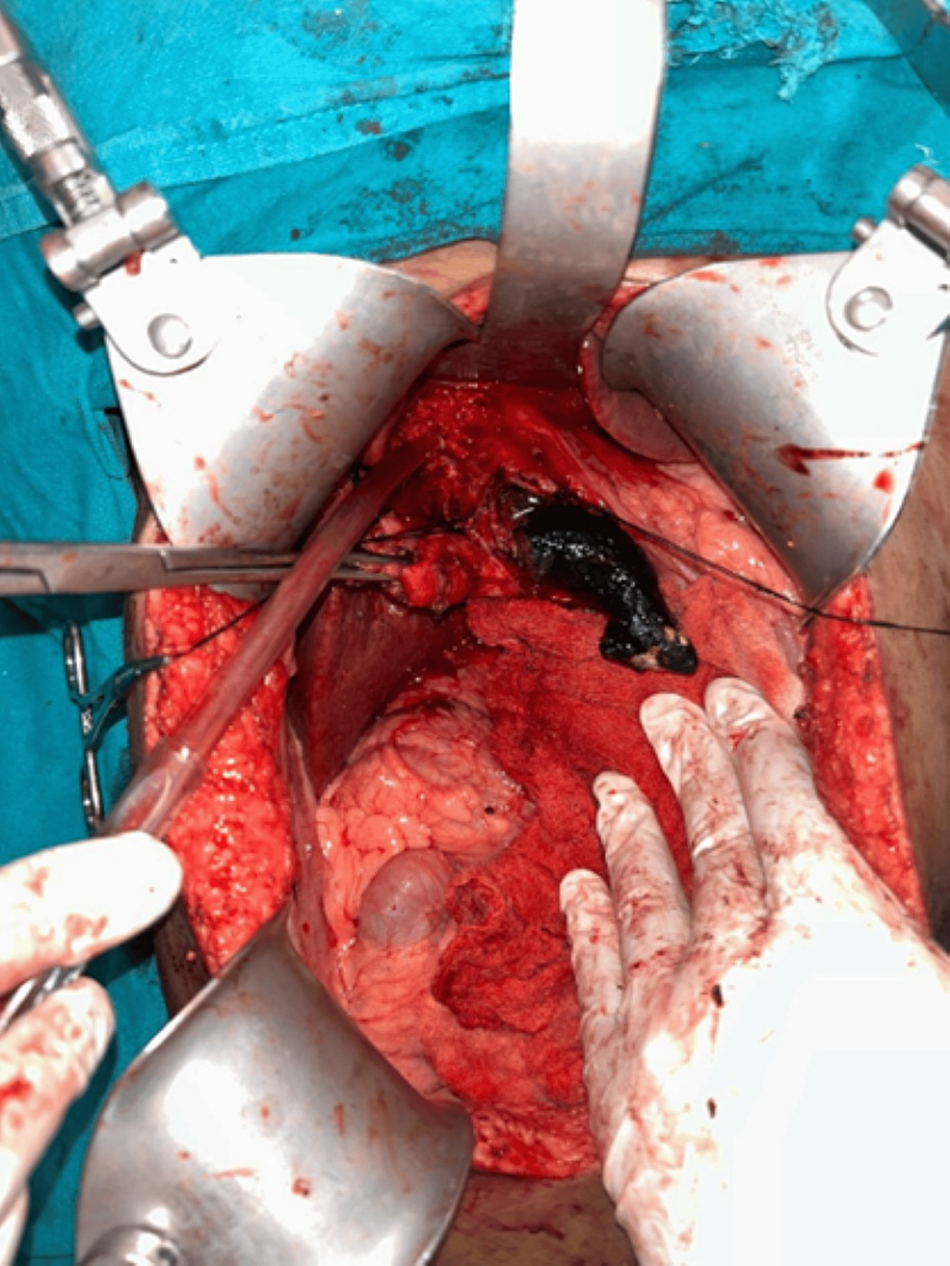
Intraoperative images showing giant common bile duct stone after anterior choledochotomy

**Figure 5:**
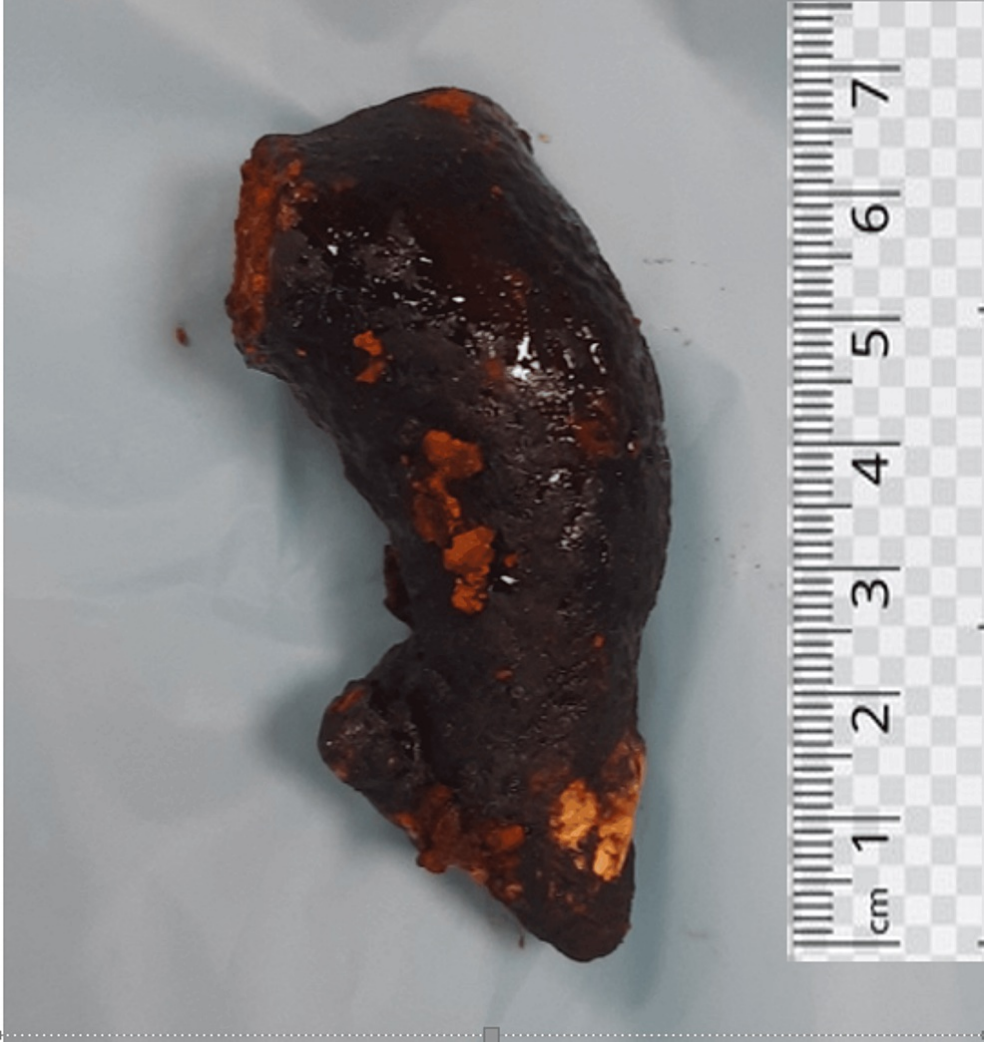
Giant common bile duct calculi of approximately 7 cm x 3 cm in size

Two stay sutures were taken over the choledochal cyst wall laterally. Bile aspirated from the cyst and sent for culture/sensitivity. Choledochotomy was made and bile was aspirated (Figure 4). Giant calculi of approximately 7 cm x 3 cm were retrieved from the CDC (Figure 5). Distal dissection of the intrapancreatic portion was done till ~5 mm proximal to the tapered end. The distal end was transacted and oversewn using PDS 4-0 suture in a double layer. The proximal end was transacted 1 cm distal to the hilum. The cyst wall was sent for histopathology. Proximal jejunum was transacted ~30 cm distal to DJ flexure using a linear cutting blue 60 mm stapler. Cut ends oversewn using prolene 4-0 in a continuous manner. Opening made in transverse mesocolon and distal jejunal limb was delivered in retrocolic fashion. Tension-free end-to-side hepaticojejunostomy was done using prolene 4-0 sutures in an interrupted fashion. A bile leak was noticed from the anastomotic site and two separate sutures were taken at the leak site. Saline wash was given and a 32 Fr abdominal drain was placed in subhepatic space. The side-to-side jejuno-jejunal anastomosis was done using double-layered anastomosis in a continuous fashion (outer layers with prolene 4-0 and inner layers with vicryl 3-0). The postoperative period of the patient was uneventful. The drain was removed on postoperative day 3 and the patient was discharged on postoperative day 7.

Histopathological examination of the gall bladder showed features of chronic cholecystitis with cholelithiasis, and a section from the CDC showed a dilated duet lined by columnar epithelium with lamina propria showed mild chronic inflammation. No evidence of dysplasia or malignancy was seen. Two reactive lymph nodes were also identified.

## Discussion

Choledocholithiasis is found in 3-10% of patients with gallstone disease [1], with some studies reporting rates as high as 14.7% [7]. The primary intervention for CBD stones is typically endoscopic retrograde cholangiopancreatography (ERCP), although other procedures like percutaneous and transhepatic stone removal, open CBD exploration, and laparoscopic approaches are also employed [8]. The occurrence of a giant CBD calculus, defined as exceeding 5 cm, is rare [9]. There are only a few documented cases of giant CBD calculi in the literature, and the presence of a giant calculus without associated jaundice is even more uncommon [2,5,6,9-18].

MRCP serves as a non-invasive technique with the potential to detect choledocholithiasis in the preoperative setting [19]. MRCP is considered the diagnostic method of choice for CBD stones, demonstrating comparable accuracy to ERCP and intraoperative cholangiography [20]. Several ERCP techniques for treatment, such as mechanical lithotripsy, endoscopic papillary large balloon dilation (EPLBD), biliary stenting, and extracorporeal shock-wave lithotripsy, can be used alone or in combination for a large CBD stone. On the other hand, large stones with a diameter ≥20 mm usually require fragmentation before extraction. In particular, huge CBD stones ≥30 mm cannot usually be captured by a basket for mechanical lithotripsy. In such cases, peroral cholangioscopy can be attempted by either one of three endoscopy methods: 1) dual operator cholangioscopy (mother-baby scope), 2) single-operator cholangioscopy (SpyGlass Direct Visualization DS, Boston Scientific, Marlborough, USA), and 3) direct cholangioscopy with an ultra-slim endoscope. ERCP with sphincterotomy is often unsuccessful for giant CBD calculi due to their size, necessitating surgical intervention through choledochotomy and stone retrieval [6].

The treatment approach for CDCs involves excision with reconstruction through bilio-enteric anastomosis. Biliary continuity is restored using either Roux-en-Y hepaticojejunostomy or hepaticoduodenostomy. In the presented case, an open cholecystectomy was performed along with the excision of the CDC and Roux-en-Y hepaticojejunostomy.

## Conclusions

Stones within the CBD can grow significantly in size, often without noticeable clinical symptoms. Surgeons should exercise caution and refrain from persisting excessively with endoscopic interventions. In summary, the extraction of a CBD stone surpassing 5 cm through endoscopic methods poses challenges, typically prompting a preference for open surgical techniques. In instances of giant CBD calculi, like the one described, it's important to note that jaundice may not always be present. The coexistence of simultaneous giant choledocholithiasis and a CDC without jaundice is a rare phenomenon.
